# Dorsolateral Prefrontal Cortex: A Possible Target for Modulating Dyskinesias in Parkinson's Disease by Repetitive Transcranial Magnetic Stimulation

**DOI:** 10.1155/2008/372125

**Published:** 2007-11-26

**Authors:** I. Rektorova, S. Sedlackova, S. Telecka, A. Hlubocky, I. Rektor

**Affiliations:** First Department of Neurology, Masaryk University, Saint Anne's Hospital, Pekařská 53, 65691Brno, Czech Republic

## Abstract

We studied whether five sessions of 10 Hz repetitive transcranial magnetic stimulation (rTMS treatment) applied over the dorsolateral prefrontal cortex (DLPFC) or the primary motor cortex (MC) in advanced Parkinson's disease (PD) patients would have any effect on L-dopa-induced dyskinesias and cortical excitability. We aimed at a randomised, controlled study. Single-pulse transcranial magnetic stimulation (TMS), paired-pulse transcranial magnetic stimulation, and the Unified Parkinson's Disease Rating Scale (UPDRS parts III and IV) were performed prior to, immediately after, and one week after an appropriate rTMS treatment. Stimulation of the left DLPFC induced a significant motor cortex depression and a trend towards the improvement of L-dopa-induced dyskinesias.

## 1. INTRODUCTION

The neural mechanisms that underlie L-dopa-induced dyskinesias in Parkinson’s disease (PD) remain poorly understood [1–4]. Dyskinesias have been associated with pulsatile stimulation of dopamine receptors, downstream changes in genes and proteins, and changes in nondopamine transmitter systems [4–6]. All these changes lead to alterations in the firing patterns within the basal ganglia, causing overactivations of the primary motor cortex and premotor cortical areas [7, 8]. Activation studies in PD patients (H_2_^15^O positron emission tomography (PET) and ^133^Xe single photon emission computed tomography (SPECT)) are in line with these results, and indicate that L-dopa-induced dyskinesias are associated with inappropriate overactivity of striatofrontal projections both at rest and during volitional actions [2, 9]. Comparison of rest with dyskinesias versus rest without dyskinesias showed a relative activation of the motor cortex, the lateral and mesial premotor areas including the supplementary motor area (SMA), the dorsal prefrontal cortex, and the basal ganglia. The comparison of dyskinetic versus nondyskinetic states during voluntary movement revealed additional activation of the same brain regions [9]. Unfortunately, in the PET study of Brooks et al. [9] mentioned above, regional cerebral blood flow (rCBF) in PD patients with and without L-dopa induced dyskinesias was not compared to that of healthy subjects, and therefore, it is difficult to speculate whether the relative increases in rCBF observed in patients with dyskinesias were in fact above or still below that of controls in all the reported areas including the DLPFC.

Repetitive transcranial magnetic stimulation (rTMS) can produce changes in excitability of cortical circuits that outlast the period of stimulation, opening the possibility of intervening directly with the mechanisms of cortical plasticity in the human cortex. rTMS is also a suitable tool to investigate plasticity within a distributed functional network since conditioning effects of rTMS are not limited to the stimulated area but give rise to functional changes in interconnected cortical areas. The magnitude and the direction of rTMS-induced plasticity depend on extrinsic factors (i.e., the variables of stimulation such as intensity, frequency, and total number of stimuli) and intrinsic factors (i.e., the functional state of the cortex targeted by rTMS). High frequencies of rTMS applied over the motor cortex lead to facilitatory aftereffects on corticospinal excitability while low-frequency stimulations lead to opposite (i.e., inhibitory) aftereffects (for review, see 10). The main problem remains, that the basic mechanisms mediating the effects of rTMS are still poorly understood.

Recenly, several studies have suggested the therapeutic efficacy of rTMS in PD while other studies have found no clinical improvement of motor performance in PD [11–15]. Using rTMS over the SMA, an Italian group [4] showed that 900 pulses of 1 Hz rTMS with an intensity set at 90% of the resting motor excitability threshold markedly reduced drug-induced dyskinesias in 8 advanced PD patients. In spite of this promising result, a year later the same group [16] discovered that 5 repeated sessions of the 1 Hz rTMS failed to enhance and/or prolong the beneficial effects of the procedure, and thus could not be considered clinically useful. Very similar results were reported by a Toronto group [17] who used the same stimulation parameters but targeted the primary motor cortex contralateral to the side with more severe dyskinesias in 6 PD patients. Although the benefit was evident at day 1 after a two-week course of rTMS, the authors have not managed to obtain more sustained clinical improvement after the treatment.

We recently reported the results of a pilot study that investigated whether 5 repeated sessions of high frequency rTMS at 10 Hz applied over the motor cortex (MC) of the relevant leg or over the left dorsolateral prefrontal cortex (DLPFC) might result in a modification of “off”-related freezing of gait and motor symptoms of PD in 6 advanced PD patients [18]. The selection of MC and DLPFC targets for rTMS was based on dopamine release in the putamen and caudate, respectively [19, 20]. The target selection was further supported by the results of Lomarev et al. [14] who had reported positive cumulative benefit of high-frequency rTMS applied over the same targets for improving gait, as well as reducing upper limb bradykinesia in PD patients. In our study, rTMS was well tolerated. Despite a reported trend towards improvement of the Stroop test interference after rTMS over the left DLPFC, freezing of gait and motor symptoms of PD remained unchanged by rTMS.

Interestingly, patients who had undergone the DLPFC treatment reported a subjective improvement of their dyskinesias. Therefore, in this paper, we present a post hoc analysis of the data from the same group of patients that focuses on cortical excitability and on the possible modification of L-dopa-induced dyskinesias by repeated sessions of rTMS over the DLPFC.

## 2. PATIENTS AND METHODS

We studied 6 patients (5 men and 1 woman, mean age 67.3±7.7 years) with advanced PD according to the UK Parkinson’s Disease brain bank criteria [21, 22], a Hoehn-Yahr stage [23] between 2.5 and 4 while in the “off” state, and without dementia (Mini-Mental State Examination [24] score >24). A Montgomery-Asberg Depression Rating Scale [25] cut-off score of 14 was used to exclude patients with depression. Patients with a predominant tremor form of PD were also excluded from the study. All patients had dyskinesias, motor fluctuations, and “off”-related freezing of gait. The disease duration was 11.3±3.1 years. The daily L-dopa dose equivalent was 1145.8±413.2 mg; all patients were on L-dopa ± entacapone plus a dopamine receptor agonist. The antiparkinsonian medication was stable for at least 4 weeks prior to the study commencement and during the study. All patients signed the informed consent form, which was approved by the Ethics Committee of Saint Anne’s Hospital in Brno.

rTMS was applied over the left DLPFC (5 cm anterior to the optimum scalp position for activation of the contralateral first dorsal interosseus muscle [26]) or over the optimal position for the tibialis anterior muscle contralateral to the foot which was most frequently used by the patient to make the first step after freezing of gait (i.e., MC), using the Magstim Super Rapid stimulator and a figure-of-eight coil with a mean diameter of 7 cm. The coil was placed tangentially to the scalp with the handle pointing backwards and laterally at 45° angle away from the midline inducing a posterior-anterior current in the brain. Nowadays, the frameless stereotaxy has been used in order to target the region of brain corresponding to cytoarchitectonic area 9/46 as defined by Petrides and Pandya [27]. Nevertheless, this part of the brain was found to overlap with the region targeted by the standard procedure (Rektorova and Paus, unpublished data), used in most studies.

rTMS was delivered in the “on” state (i.e., when PD medication was working and providing benefit) without dyskinesias according to the individual patients’ diaries, at the same time of day. One treatment consisted of 5 sessions over 5 consecutive days. We chose a repeated sessions design, and our patients were stimulated while being in their “on” state since both conditions have been associated with better rTMS outcomes in literature [12, 14]. One session consisted of 1350 pulses delivered at 10 Hz frequency, at an intensity of 90% of the resting motor excitability threshold (for the right first dorsal interosseus muscle when stimulating the left DLPFC, and the appropriate tibialis anterior muscle when stimulating the MC). Each patient completed at least one rTMS treatment. The order of stimulation sites was randomized using closed envelopes. Although we aimed at a crossover design, only two patients completed both treatments (with a one-month interval between them).

In order to ensure the method was safe with regard to cognitive functions, a brief neuropsychological battery of tests was administered prior to, immediately after, and one week after each rTMS treatment. The results have been published elsewhere [18].

Experienced neurologists who were blinded to the stimulation site performed a neurological evaluation prior to, immediately after, and one week after appropriate rTMS treatment. The evaluation was conducted during the patients’ relative “off” state (i.e., when PD medication was not improving PD symptoms resulting in a lack of mobility) according to the individual patients’ diaries, at the same time of day. It consisted of the UPDRS, part III (motor examination) and part IV (treatment complications) [28]. A more detailed evaluation and analysis of gait and “off”-related freezing of gait has been reported elsewhere [18].

Single-pulse and paired-pulse TMS was performed while the patient was “on” to study the effects of rTMS treatments on cortical excitability. We used the paired-pulse TMS paradigm of Ridding et al. [29] at interstimulus intervals (ISI) of 1, 3, 7, and 15 milliseconds [30]. We evaluated motor evoked potentials (MEPs) elicited by single and paired-pulse TMS pulses over the MC contralateral to either the right first dorsal interosseus muscle (prior to, immediately after, and one week after the rTMS treatment over the left DLPFC) or the appropriate tibialis anterior muscle (prior to, immediately after, and one week after the rTMS treatment over the appropriate MC). The motor cortical sites were chosen to specifically accommodate the primary goal of our freezing of gait protocol design. A circular coil was used. MEP size reflects more globally the corticospinal input-output balance, excitatory inputs from high-threshold glutamatergic pathways to the motor cortex lead to intracortical facilitation, whereas inhibitory inputs from low-threshold GABA-A-mediated pathways lead to intracortical inhibition at short ISI [13].

## 3. STATISTICAL ANALYSIS

The effects of rTMS treatments on single MEP amplitudes and UPDRS before and after the rTMS treatments were analysed by a two-tailed paired-sample *t* test. A two-factor repeated measures ANOVA, with the factors of “TIME” (3 levels: baseline, immediately after the rTMS treatment, and 1 week after the rTMS treatment) and “ISI” (3 levels: averaged data from the inhibiting ISI (1 and 3 milliseconds), the facilitating ISI (15 milliseconds), and the intermediate ISI (7 milliseconds)) was performed to evaluate the effects of the rTMS treatment applied over the MC and the DLPFC, respectively.

## 4. RESULTS

### 4.1. Clinical effects

Despite our aim to perform a randomised crossover design, the study was stopped prematurely as several patients withdrew the consent form before completing both treatments. A subjective lack of treatment effect on freezing of gait (i.e., the primary outcome) was the reason for the study withdrawal. This study presents the results from four patients that completed the rTMS treatment over the MC and four patients that completed the treatment over the DLPFC. Despite the lack of effect of rTMS on freezing of gait and motor symptoms of PD [18], the patients reported a decreased frequency and intensity of dyskinesias after 5 consecutive sessions of rTMS targeted over the left DLPFC. These changes were reflected by the mean UPDRS IV decrease immediately after 5 sessions of the stimulation as compared with the baseline scores (see Table 1). The result did not reach statistical significance (P=.06) but could be suggestive of a mild improvement of dyskinesias. No such changes were observed after rTMS of the MC.

### 4.2. Effects on cortical excitability

There was no cumulative effect of the rTMS treatment over the MC on corticospinal excitability as measured by MEP responses produced by single TMS pulses over the left MC contralateral to the appropriate tibialis anterior muscle (see Table 2). However, we found that 5 consecutive sessions of high frequency rTMS of the left DLPFC produced a significant decrease in the amplitude of MEP responses produced by single TMS pulses over the left MC contralateral to the right first dorsal interosseus muscle (see Table 2).

There were no effects of any rTMS treatments on the time course of intracortical inhibition or intracortical facilitation in the appropriate motor cortex; this was verified with a two-factor repeated measures ANOVA with the factors “TIME” and “ISI”: F(4,26) =0.72, P=.6 for rTMS treatment over the DLPFC; F(4,10) =2.4, P=.1 for rTMS treatment over the MC.

## 5. DISCUSSION

The results of our study point to a possible impact of repeated sessions of rTMS applied over the left DLPFC on L-dopa-induced dyskinesias in advanced PD patients. The mean change in the UPDRS IV scores did not reach a statistical significance, but we did not evaluate dyskinesias by more appropriate standardized scales since the assessment of dyskinesias was not the primary outcome of our pilot study. On the other hand, UPDRS IV was used to test dyskinesias in a PET study by Brooks et al. [9]. There are a number of hypothetical mechanisms that might underlie the possible effects of rTMS of the DLPFC on L-dopa-induced dyskinesias in advanced PD.

It appears that changes of the excitability of the primary motor cortex may be more efficiently performed through stimulation of premotor and/or other more anterior brain regions, for review, see [31]. We found that 5 consecutive sessions of high frequency rTMS of the left DLPFC produced a significant decrease in the amplitude of MEP responses produced by single TMS pulses over the left MC for the right first dorsal interosseus muscle (i.e., decrease of the cortico-spinal excitability). These results are in accord with the results of Rollnick et al. [32] who also demonstrated that stimulation of the DLPFC via subthreshold 5 Hz rTMS induces motor cortex depression in healthy subjects. It was posited by the authors that this finding might be explained by the fact that prefrontal brain areas contribute to motor cortex inhibition through antagonisms between frontal and parietal lobes [32, 33] but the exact mechanisms are not fully understood. Primary motor cortex “overactivity” in rather advanced PD patients was found by means of PET and functional magnetic resonance imaging (fMRI) while studying simple or complex motor hand tasks [34, 35]. It was also observed in SPECT and PET studies of PD patients with L-dopa-induced dyskinesias [2, 9]. Electrophysiological data furhter support this notion: increased MEP aplitudes at rest were found in PD patients with L-dopa induced dyskinesias [36, 37]. Neuroimaging studies have shown that subthalamic nucleus stimulation acts through the reduction of abnormal overactivity in the motor system at rest [34, 38]. Even though dyskinesias were not systematically monitored in these studies, it has been well known that subthalamic nucleus stimulation surgery is able to reduce L-dopa-induced dyskinesias [39]. Taken together, normalizing (i.e., reducing) the excitability of the primary motor cortex, for example, by rTMS of the DLPFC might be one possible factor in improving L-dopa-induced dyskinesias in advanced PD patients.

rTMS applied over the left DLPFC can induce significant increases of rCBF in the stimulated area [40]. Functional imaging studies (PET and fMRI) report decreases in rCBF and MRI signal in the DLPFC and the rostral SMA in advanced PD patients compared to controls [35 41–45]. The DLPFC is connected with the rostral SMA [46]. Pallidotomy [44] and subthalamic nucleus stimulation [47] reversed such a hypoactivation. After unilateral pallidotomy, glucose metabolism increased in the DLPFC, in addition to metabolic changes in other cortical and subcortical areas [48]. rCBF increases were reported in the DLPFC and the rostral SMA during apomorphine pumps [49]. Again, pallidotomy, subthalamic nucleus stimulation and continuous apomorphine are able to reverse dyskinesias [39 49–51]. Therefore, the direct involvement of the DLPFC and the degeneration of mesofrontal dopaminergic afferents might also be hypothesized to play some role in the development of dyskinesias.

Finally, involvement of the dorsolateral “prefrontal” circuit may well be possible. The prefrontal circuit encompasses the dorsal prefrontal cortex, the dorsal caudate nucleus, the dorsal medial globus pallidus, and the ventral anterior nucleus of the thalamus. Strafella et al. have shown that high frequency rTMS of the DLPFC leads to dopamine release in the ipsilateral caudate nucleus [19, 20]. We used the same parameters as Strafella et al. with regard to frequency and intensity of stimulation. Therefore, subcortical mechanisms cannot be excluded either.

Taken together, we have shown for the first time that repeated sessions of subthreshold 10 Hz rTMS of the DLPFC induced significant reductions of the ipsilateral motor cortex excitability in advanced PD population, and might possibly be suggestive of an improvement of L-dopa-induced dyskinesias. It has to be pointed out that this was a small observational study not specifically designed to assess dyskinesias in PD. DLPFC stimulation was not controlled by the placebo rTMS and therefore a possible placebo effect cannot be excluded either [15]. On the other hand, the DLPFC stimulation was controlled by yet another active stimulation over the MC. Improvement of dyskinesia could not have been expected by patients since changes in the motor complications score were not included in the primary outcomes of our pilot study.

Further research is warranted to explore more precisely whether the unilateral or bilateral stimulation of the DLPFC via repeated sessions of high frequency rTMS induces beneficial effects on L-dopa-induced dyskinesias in advanced PD patients, and whether it could be considered clinically useful.

## Figures and Tables

**Table 1:** Effects of rTMS treatments on motor symptoms of PD and treatment complications as assessed by the UPDRSs III and IV. UPDRS III: Unified Parkinson’s Disease Rating Scale, part III, motor examination; UPDRS IV: Unified Parkinson’s Disease Rating Scale, part IV, treatment complications; Pre-rTMS/MC: prior to the repetitive transcranial magnetic stimulation treatment applied over the motor cortex; Post-rTMS/MC: immediately after the repetitive transcranial magnetic stimulation treatment applied over the motor cortex; Pre-rTMS/PFC: prior to the repetitive transcranial magnetic stimulation treatment applied over the prefrontal cortex; Post-rTMS/PFC: immediately after the repetitive transcranial magnetic stimulation treatment applied over the prefrontal cortex.

	Pre-rTMS/MC scores	Post-rTMS/MC scores	Pre-rTMS/PFC scores	Post-rTMS/PFC scores
UPDRS III “on”	21.25±13.43	21.75±13.67	20.75±12.87	20.00±14.12
UPDRS IV	6.00±1.83	7.00±2.50	6.25±1.71	4.75±0.96

**Table 2:** Effects of rTMS treatments on the amplitude of MEP responses produced by single TMS pulses. MEP: motor evoked potential; Pre-rTMS/MC: prior to the repetitive transcranial magnetic stimulation treatment applied over the motor cortex; Post-rTMS/MC: immediately after the repetitive transcranial magnetic stimulation treatment applied over the motor cortex; Pre-rTMS/PFC: prior to the repetitive transcranial magnetic stimulation treatment applied over the prefrontal cortex; Post-rTMS/PFC: immediately after the repetitive transcranial magnetic stimulation treatment applied over the prefrontal cortex.

	Pre-rTMS/MC	Post-rTMS/MC	Pre-rTMS/PFC	Post-rTMS/PFC
MEP amplitude μV)	1.60±0.79	1.52±0.68	${2.02\pm 0.10}^{*}$	${1.22\pm 0.32}^{*}$

${}^{*}P=.03$ (paired samples *t* test).
